# Advances in the application of multi-omics analysis in skin aging

**DOI:** 10.3389/fragi.2025.1596050

**Published:** 2025-07-31

**Authors:** Boquan Long, Weitian Pan, Shuozhong Wu, Qianye Nong, Wenhui Li, Siqi Chen, Hongwei Guo

**Affiliations:** ^1^ Dermatology Department of the Second Affiliated Hospital of Guangdong Medical University, Zhanjiang, China; ^2^ The First Clinical College, Guangdong Medical University, Zhanjiang, Guangdong, China

**Keywords:** skin aging, multi-omics, biomarkers, pathogenesis, anti-aging

## Abstract

Skin aging is a progressive decline in the structural integrity and physiological function of the skin, driven by a complex interplay of intrinsic and extrinsic factors. Consequently, skin aging is classified into intrinsic and extrinsic aging. Intrinsic aging is characterized by epidermal thinning, dryness, fine lines, and reduced elasticity over time, whereas extrinsic aging manifests as epidermal thickening, deep wrinkles, skin laxity, roughness, and pigmentation, particularly in sun-exposed areas, such as the face, neck, and hands. The underlying mechanisms of these two aging processes are intricate and distinct, encompassing various elements, including temporal aspects, genetic predisposition, immune responses, endocrine influences, and ultraviolet radiation. Multi-omics approaches—including macro-genomics, epigenetics, transcriptomics, proteomics, and metabolomics—offer valuable insights into the mechanisms and pathogenesis of skin aging while aiding in the identification of biomarkers and potential therapeutic targets. This review provides an overview of advancements in skin aging research using multi-omics technologies, aiming to foster innovation in research methodologies related to skin aging.

## 1 Introduction

**FIGURE 1:**
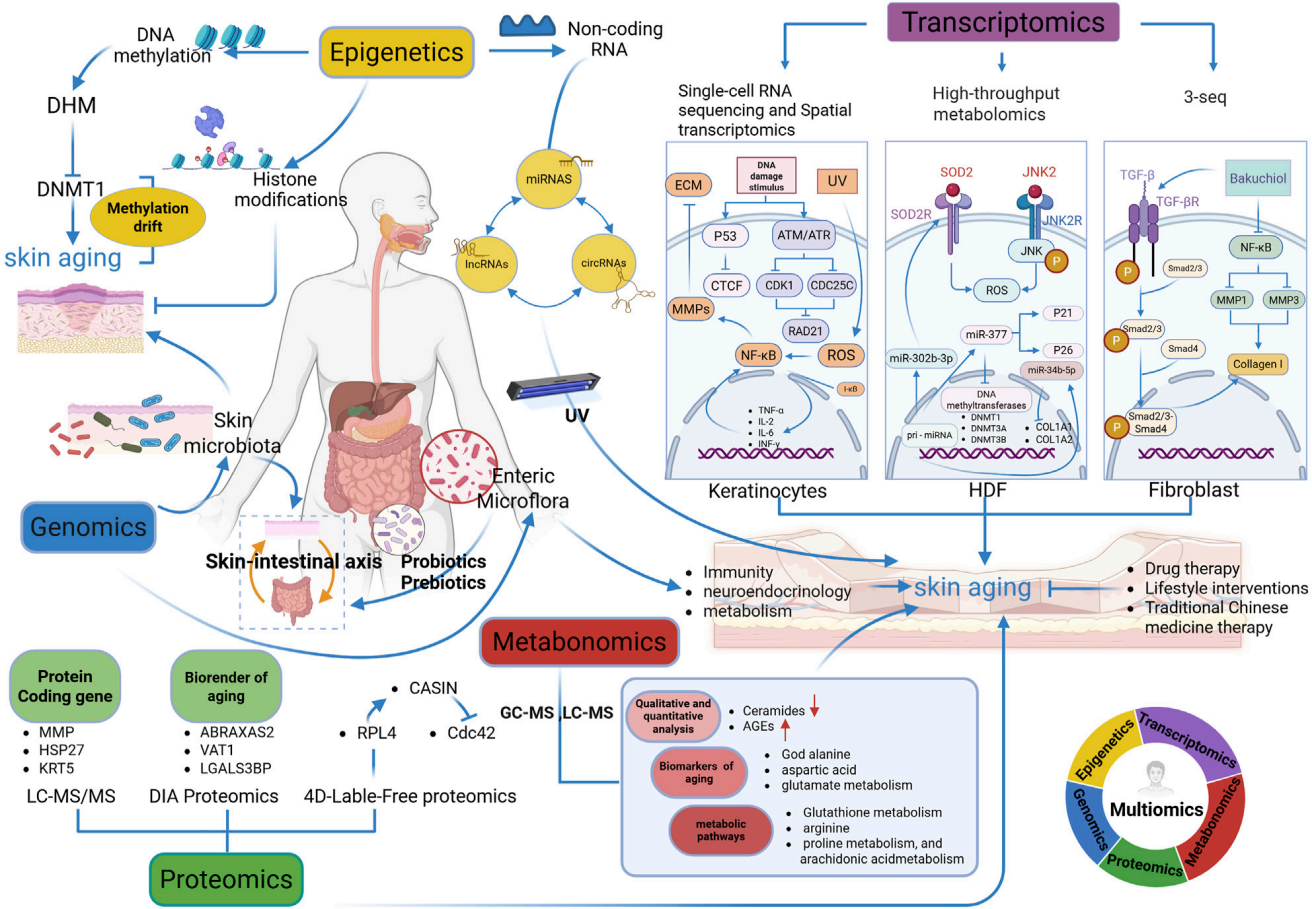
Multi-omics analysis in skin aging research (Figure created using Biorender. com, accessed on 5 March 2025). Multi-Omics Integration: Rainbow Circle (Bottom-right Corner). The circular diagram shows the cross-integration of genomics (dark blue), epigenetics (yellow), transcriptomics (purple), metabonomics (red) and proteomics (green), reflecting the research idea of multi-omics collaboration in analyzing skin aging. DHM, dihydromyricetin; DHMT1, DNA methyltransferase 1; 3-seq, 3-end sequencing expression quantification; CTCF, CCCTC binding factor; ATM: ataxia telangiectasia mutated; ATR, ataxia telangiectasia and Rad3-related protein; CDK1, cyclin-dependent kinases 1; CDC25C, cell division cycle 25C; RAD21, RAD21 cohesin complex component; ROS, reactive oxygen species; ECM, extracellular matrix; TNF-α, tumor necrosis factor-alpha; IL-2, interleukin-2; IL-6, interleukin-6; INF-γ, interferon-γ; HDF, human dermal fibroblasts; SOD2, superoxide dismutase 2; JNK, c-Jun N-terminal kinase; DNMT3A, DNA methyltransferase 3A; DTNMT3B, DNA methyltransferase 3B, COL1A1: collagen type I alpha 1 chain; COL1A2, collagen type I alpha 2 chain; LC-MS/MS, Liquid Chromatography-Tandem Mass Spectrometry; DIA Proteomics, Data-Independent Acquisition Proteomics; 4D-Label-Free Proteomics, Four-Dimensional Label-Free Quantitative Proteomics; MMP, matrix metalloproteinases; MMP1, matrix metalloproteinases 1; MMP2, matrix metalloproteinases 2; MMP3, matrix metalloproteinases 3; HSP27, heat shock protein 27; KRT5, keratin 5; ABRAXAS2, Abraxas 2, BRISC complex subunit; VAT1, vesicle amine transport 1; LGALS3BP, lectin galactoside-binding soluble 3 binding protein; RPL4, 60S ribosomal protein L4; CDC42, cell division control protein 42 homolog; CASIN, Cdc42 activity-specific inhibitor; GC-MS, gas chromatography-mass spectrometry; LC-MS, liquid chromatography-mass spectrometry; AGEs, advanced glycation end products Remark: →, promote; ┤, inhibit.

Aging is a progressive and irreversible pathophysiological process characterized by the deterioration of tissue and cellular function, accompanied by an increased risk of various age-related disorders. With the evolution of the social economy and the growing demand for beauty, skin aging has become a key area of research. However, its underlying mechanisms is intricate, and research methodologies face limitations. In recent years, the implementation and advancements in multi-omics technologies have enhanced and broadened the scope of skin aging research, extending from macro-genomic, epigenetic, and transcriptional dimensions to protein and metabolic levels. This review explores advancements in multi-omics technologies—including macro-genomics, epigenetics, transcriptomics, proteomics, and metabolomics—in the context of skin aging, aiming to provide new perspectives and strategies to advance research methodologies in this field (Figure 1).

## 2 Macro-genomics

**FIGURE 2:**
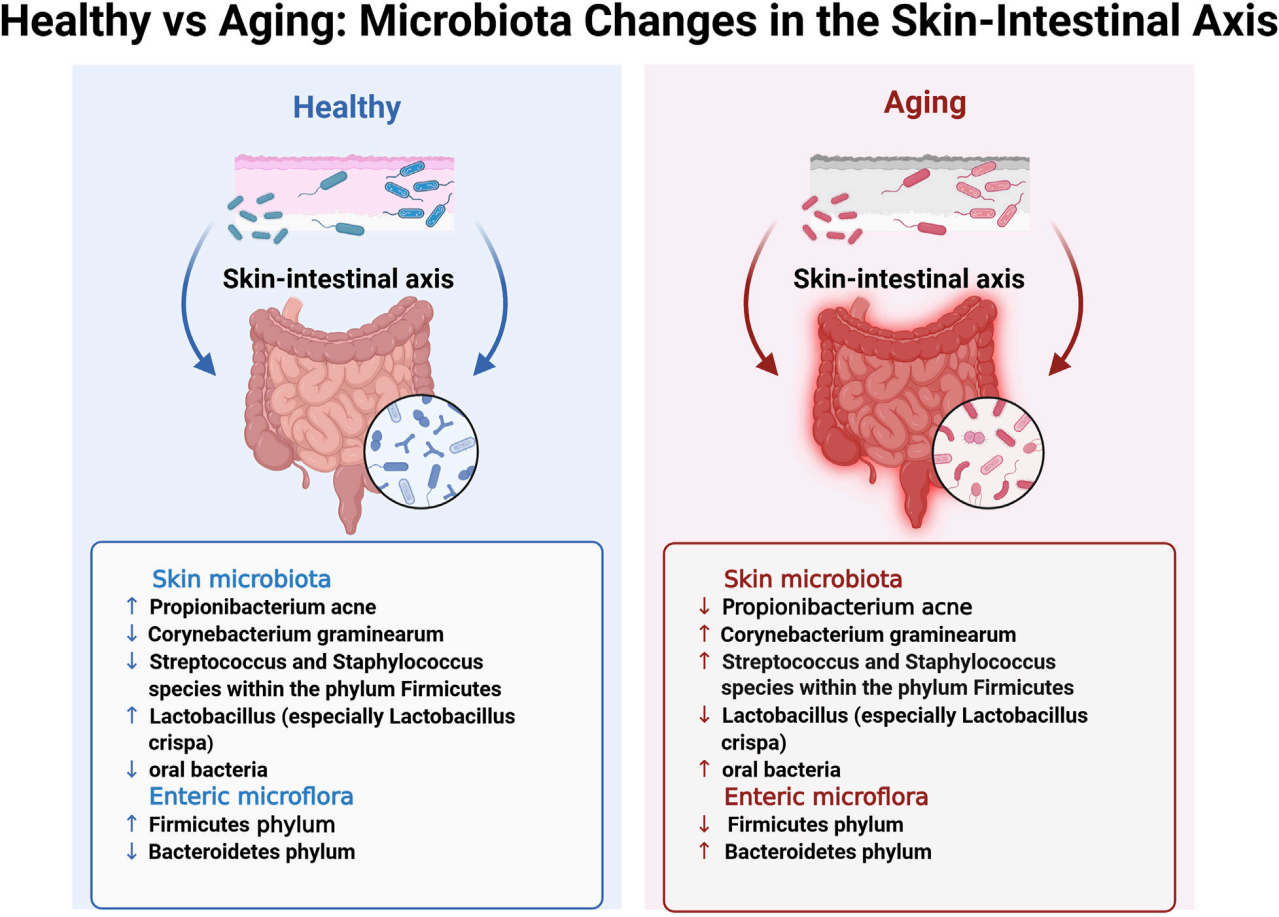
Healthy vs. Aging: Microbiota Changes in the Skin-Intestinal Axis. This figure provides a detailed illustration of microbiota alterations in the skin–intestinal axis observed between healthy and aging individuals, corresponding to the highlighted blue box in the lower-left corner of Figure 1. The bidirectional blue and red arrows intuitively represent the functional interactions and pattern changes of the skin-intestinal axis in the healthy and aging states, respectively. Remark: ↑, higher levels in specified microbial taxon when compared with aging/healthy; ↓, lower abundance in specified microbial taxon when compared with aging/healthy.

Macro-genomics is a scientific discipline that focuses on the analysis of the genomes of microbial communities within environmental contexts. In contrast to traditional culture methods, macro-genomics utilizes high-throughput sequencing technology to directly analyze all microbial genomes in environmental samples, providing more extensive and precise information. In skin research, macro-genomics elucidates the structural and functional characteristics of the skin and gut microbiota, offering valuable insights into microbial alterations associated with skin aging (Figure 2).

### 2.1 Skin microflora

Garlet et al. (2024) conducted whole-genome sequencing analyses on facial skin swabs from 100 healthy Caucasian females (≥18 years) to examine age-related alterations in skin flora. The results revealed that older females (≥55 years) exhibited greater microbial diversity, characterized by a marked decrease in the abundance of actinomycetes, such as *Propionibacterium acnes*, and a substantial increase in the number of *Corynebacterium graminearum* compared to the younger group (18–35 years) (Figure 2). Additionally, there was an increase in the abundance of *Streptococcus* and *Staphylococcus* species within the phylum Firmicutes, as well as an increase in the abundance of species from the phylum Aspergillus. In contrast, the younger age group showed a higher prevalence of *Propionibacterium acnes* and *Lactobacillus*, particularly an increased abundance of *Lactobacillus crispa*. This increase correlated with elevated expression levels of genes associated with metabolic processes and immune defense in the skin flora compared to those in the older cohort. These findings suggest that the skin flora of younger individuals may exhibit enhanced immune protection and metabolic activity. Furthermore, Woolery-Lloyd et al. identified substantial age-related variations in the skin microbiome (Woolery-Lloyd et al., 2023). Their study revealed that healthy older Japanese females (aged 60–76) exhibited a less diverse skin microbiome than younger females (aged 21–37). However, microbial diversity was higher on the forehead, cheeks, and forearms of older individuals, with a notable increase in the abundance of oral bacteria. The age-related decrease in *Propionibacterium* abundance may contribute to the increased microbial diversity observed in older females. Particularly, post-menopausal reductions in sebum secretion, coupled with reduced *Propionibacterium* abundance, may facilitate the proliferation of various bacterial species, thereby increasing the likelihood of skin inflammation, including certain types of dermatitis (Khmaladze et al., 2020). Overall, the skin microbiota may expedite the skin aging process by regulating immune responses, affecting oxidative stress levels, and impairing the skin barrier, among other functions.

### 2.2 Intestinal microflora

In contrast to the skin microbiota, the gut microflora may influence skin aging through intricate mechanisms involving immunological, neuroendocrine, and metabolic pathways. Bock et al. (2024) reported that most species residing on the mucus surface of the gut or within the intestinal lumen belonged to four principal phyla: Firmicutes, Bacteroidetes, Actinomycetes, and Ascomycetes. The gut microbiota plays a crucial role in various synthetic processes, including the production of vitamins, amino acids, short-chain fatty acids (SCFAs), and other metabolites, through enzymatic activity and various metabolic pathways. SCFAs, including butyrate, propionate, and acetate, are byproducts of fiber fermentation by gut microbiota and have demonstrated anti-inflammatory and immunomodulatory properties (Boyajian et al., 2021). Research suggests that as individuals transition from adulthood to old age, the relative abundance of the Firmicutes phylum decreases, with a concurrent increase in the abundance of the Bacteroidetes phylum, resulting in a decreased ratio between the Firmicutes phylum and Bacteroidetes phylum (F/B ratio). The F/B ratio has been demonstrated to be crucial in SCFA synthesis. Notably, age-related microbial dysregulation may expedite aging, promote inflammation and frailty, and negatively affect overall health and longevity (Ratanapokasatit et al., 2022). A comparative analysis of intestinal microbiota between residents of a longevity village and urbanized older adults revealed that the former exhibited higher levels of *Bacteroides fragilis*, whereas the latter had a greater proportion of *Bacteroides mimosoides*. Additionally, *Lactobacillus* species exhibited greater diversity among individuals from the longevity village. Microbiological studies on centenarians with a low prevalence of chronic diseases and prolonged healthy lifespan have demonstrated that their bacterial diversity exceeds that of older adults in the same region and even surpasses that of the general adult population. Bacterial species associated with immunological and metabolic health were more prevalent among centenarians, including taxa from Ascomycetes, Actinobacteria, and Verrucomicrobia, as well as the genera Akkermansia, Christensenellaceae, and other *Lactobacillus* species (Ratanapokasatit et al., 2022; Woo and Kim, 2024). The relationship between gut and skin microbiota has become a significant research focus, highlighting a bidirectional communication pathway known as the gut-skin axis, which links the skin microbiota to the enteromembrane system. Substantial evidence also supports the concept of a skin-gut axis. Studies have demonstrated that increased intestinal permeability owing to gut microbial dysbiosis results in the accumulation of bacterial metabolites, such as phenolic compounds derived from aromatic amino acids, in the skin, thereby affecting epidermal differentiation and compromising skin integrity (Mahmud et al., 2022). Although the precise mechanisms by which the gut microbiota influences the skin through the immune system remain elusive, future research is expected to provide deeper insights.

### 2.3 Probiotics and prebiotics in combating skin aging via the gut-skin axis

Recent research suggests that probiotics and prebiotics play a beneficial role in slowing skin aging, given the significant correlation between the gut flora and healthy aging. Woolery-Lloyd et al. (2023) found that oral probiotics and prebiotics improved stratum corneum hydration, enhanced photoprotection, and reduced wrinkle depth and photoaging. A randomized, double-blind, placebo-controlled trial involving 110 participants aged 41–59 years demonstrated significant improvements in skin hydration, wrinkle reduction, radiance, and elasticity after 12 weeks of *Lactobacillus* yorkii (HY7714) treatment. HY7714 extracellular polysaccharides modulate intestinal tight junctions in human intestinal adenocarcinoma cells (Caco-2) by upregulating the expression of genes for occludin-1 and zonula occludens-1. This regulation influences the gut-skin axis and modifies dermal cellular characteristics, indicating that the probiotic aids in restoring the equilibrium between free radical production and elimination (Ratanapokasatit et al., 2022; Woo and Kim, 2024). A randomized, double-blind, placebo-controlled trial involving 600 healthy Japanese adult females demonstrated that daily intake of *Bifidobacterium shortum* and oligogalactose from probiotics for four consecutive weeks effectively improved skin hydration and promoted histone L activity, a crucial marker of keratinocyte differentiation. Additionally, the active group exhibited significant reductions in serum and urinary phenolics (Ratanapokasatit et al., 2022).

Moreover, the topical application of probiotics, prebiotics, and postbiotics has been demonstrated to enhance wrinkle appearance, age-related hyperpigmentation, and skin moisture. A preliminary pilot study (Woo and Kim, 2024) demonstrated enhancements in wrinkle depth and severity, as well as a reduction in forehead and interbrow hyperpigmentation, following 7 days of application of a high-dose anti-inflammatory probiotic facial spray. The active ingredient in this facial mist, Eukaryotic Nitrosomonas, is a non-pathogenic bacterium that exerts anti-inflammatory effects by converting ammonia enzymes in sweat into nitrite and nitric oxide. Additionally, a separate study demonstrated that *Lactobacillus* Tyndallized KCCM12625P (AL) markedly reduced ultraviolet B (UVB)-induced increases in reactive oxygen species (ROS) levels, as evidenced by H_2_DCFDA staining analysis. These findings highlight the antioxidant properties of AL, establishing its efficacy in wrinkle inhibition and prevention (Yu et al., 2022; Jang et al., 2024).

## 3 Epigenetics

Epigenetics is a significant subdiscipline of genetics that investigates heritable changes in gene expression or cellular phenotype resulting from the modification of specific processes without DNA sequence alteration. Principal epigenetic mechanisms include DNA methylation, histone modification, chromatin remodeling, and non-coding RNA control. Among these, DNA methylation is the most extensively and well-researched form of epigenetic alteration.

### 3.1 DNA methylation

Jones et al. (2015) identified considerable differences in DNA methylation levels in skin samples from both young and older individuals, as well as between sun-exposed and non-exposed areas. These variations in DNA methylation were observed to correlate with the degree of skin damage at specific gene loci. This finding suggests that localized alterations in DNA methylation, whether caused by aging or external damage, may serve as predictors for aging phenotypes or certain skin disorders. Moreover, Grönniger et al. (2024) reported that age-associated changes in DNA methylation are driven by two interconnected phenomena: epigenetic drift and the epigenetic clock. To date, four epigenetic clocks have been established to assess biological age in human tissues. The Horvath clock is the most commonly used method for estimating biological age, relying on methylation levels at 353 specific CpG sites in the human genome. These sites were identified through time-series age regression analysis of approximately 27,000 DNA methylation markers. The clock exhibited an average error of 3.6 years in estimating the actual age of various tissues and cell types, demonstrating accuracy comparable to—or in some cases exceeding—that of existing forensic age prediction techniques (Gerasymchuk et al., 2020; Moltrasio et al., 2022). Further research highlights the crucial role of DNA methyltransferase 1 (DNMT1) in the epigenetic regulation of biological processes by preserving CpG site methylation. However, DNMT1 activity declines with age, leading to a significant decrease in the overall methylation levels, a phenomenon known as methylation drift in senescent cells. Recent studies have indicated that DNMT1 expression diminishes with increasing passages in senescent fibroblasts, and its silencing in young fibroblasts can induce a senescent phenotype (Moltrasio et al., 2022). Similarly, another study showed that DNMT1 deletion leads to genomic instability and cell death in both murine and human cells. The newly identified small-molecule compound GSK3685032 has demonstrated remarkable *in vivo* tolerance as a DNMT1 inhibitor, indicating a superior safety profile for selective active site inhibitors targeting DNMT1 (Gerasymchuk et al., 2020; Moltrasio et al., 2022; Falckenhayn et al., 2024). This finding indicates that identifying suitable DNMT1 inhibitors with moderate DNA methylation inhibition could facilitate skin rejuvenation. A recent study (Soheilifar et al., 2022) highlighted dihydromyricetin (DHM) as the first epigenetic inhibitor capable of inducing skin rejuvenation in humans. Notably, within just 3 days of DHM exposure in primary human keratinocyte cultures, a two-year decrease in DNA methylation age was observed. In human skin models, DHM exhibited a strong regenerative capacity and significant antiaging effect. Although the long-term rejuvenating potential of DHM remains unverified in clinical trials, it demonstrates significant promise for advancing antiaging cosmetics and synergistic applications with nutritional supplements.

### 3.2 Non-coding RNA

Several studies have demonstrated that non-coding RNAs significantly contribute to the skin aging process. Among these, microRNAs (miRNAs), long-chain non-coding RNAs (lncRNAs), and circular RNAs (circRNAs) have been identified as key contributors to UV-induced skin aging. Li et al. (2021) showed that miRNAs modulate the skin’s response to ultraviolet radiation (UVR) and healing mechanisms by regulating gene expression, participating in signaling networks, and influencing apoptosis and aging. Additionally, miRNAs play a crucial role in preserving skin barrier function. For instance, epidermally produced miR-203 modulates calcium-induced keratinocyte differentiation by activating the protein kinase C and activator protein 1 (AP-1) pathways (Lee et al., 2021). Moreover, miR-25, a specific miRNA, can suppress the expression of microphthalmia-associated transcription factor (MITF), a crucial transcription factor in melanogenesis, thereby modulating the process of melanogenesis. Additionally, miR-21-5p indirectly influences MITF activity by targeting SRY-box transcription factor 5, whereas miR-434-5p targets tyrosinase and hyaluronidase, thereby affecting melanin synthesis and transport (Moltrasio et al., 2022). Moreover, miR-21 plays an important role in managing dermatological conditions, including acne and wound healing (Tang et al., 2022). Additionally, lncRNAs have been demonstrated to participate in the photoaging process by modulating pathways involved in melanogenesis, inflammatory response, and extracellular matrix (ECM) breakdown. A study revealed notable alterations in lnc-CD1D-2 expression in primary melanocytes after UVB exposure, indicating that UVR-induced ROS formation facilitates lnc-CD1D-2 upregulation, thereby enhancing melanin synthesis (Li et al., 2021). Furthermore, Tang et al. demonstrated that lncRNAH19 may participate in photodamage by interacting with miR-296-5p to upregulate the expression of insulin-like growth factor 2, activate the phosphoinositide 3-kinase (PI3K)/mechanistic target of rapamycin pathway, and increase aquaporin 3 (AQP3) expression, ultimately preventing human dermal fibroblast (HDF) senescence and affecting skin photoaging (Tang et al., 2023). Similarly, lncRNAPVT1 may suppress the activation of the extracellular signal-regulated kinase/p38 mitogen-activated protein kinase signaling pathway by upregulating AQP3 expression through the sequestration of miR-551b-3p, thereby enhancing HDF viability, inhibiting HDF senescence, and ultimately delaying skin photoaging (Pozzo et al., 2024). Simultaneously, emerging data suggest that circRNAs exhibit differential expression in photoaged skin. Liang et al. (2024) demonstrated variations in the expression of 29 circRNAs between UV-irradiated HDF cells and their non-irradiated counterparts. UV irradiation significantly downregulated the expression of circ-COL3A1-859267. Notably, circCOL3A1-859267 functioned as a sponge for miR-29c, which plays a role in modulating the expression of type I collagen in skin fibroblasts. The downregulation of circCOL3A1-859267 expression resulted in the overexpression of miR-29c, thereby inhibiting collagen synthesis. Similarly, another study identified 39 and 89 circRNAs with upregulated and downregulated expression, respectively, in UV-treated HDFs. Using RNA sequencing (RNA-seq) technology, these circRNAs were demonstrated to be intricately associated with ECM composition and metabolic processes. Further validation through quantitative PCR confirmed the downregulation of circRNA-0006766 and circRNA-0011129 expression, indicating that circRNAs may influence aging-related pathways through miRNA absorption (Si et al., 2019).

### 3.3 Histone modifications to delay skin aging

In addition to DNA methylation, histone modifications also play a role in the skin aging process by regulating epidermal cell division, proliferation, and differentiation. Research suggests that histone modifications influence epidermal differentiation at a minimum of two key stages. First, they facilitate the transition from a quiescent to a proliferative state by influencing cell cycle inhibitor genes, transcription factors (TFs), and associated signaling pathways. Second, inhibitory histone alterations directly affect the promoter regions of differentiation-inducing genes in undifferentiated keratinocytes (Falckenhayn et al., 2024). Notably, some studies have demonstrated that the deletion of specific histone-modifying enzymes (Sen et al., 2008; Sobiak and Leśniak, 2020) does not lead to widespread activation or repression of the expression of differentiation-related genes. This suggests that distinct histone modifications may selectively regulate the expression patterns of individual genes or gene clusters rather than universally controlling the entire differentiation process. Further research is required to investigate the effects of histone modifications on skin aging.

## 4 Transcriptomics

Transcriptomics is an essential tool for analyzing the types and quantities of all RNA molecules, including miRNAs and ncRNAs, within cells or tissues. It aids in identifying functional elements of the cellular or tissue genome and uncovering molecular components, thereby enhancing the understanding of disease mechanisms in biological processes. Transcriptomics has progressed from microarray technology to high-throughput measurement methods, with RNA-seq serving as a leading method owing to its ability to provide comprehensive and precise gene expression data (Osborne et al., 2012).

### 4.1 Single-cell RNA sequencing and spatial transcriptomics

#### 4.1.1 Single-cell RNA sequencing and the advancement of spatial transcriptomics

The advent of high-throughput next-generation sequencing has driven significant progress in genomic technologies, including single-cell RNA sequencing (scRNA-seq). scRNA-seq enables the identification of rare cell populations that may be overlooked in large-scale studies and allows for precise gene expression analysis of specific cell types. However, scRNA-seq has notable limitations, primarily the inability to capture essential spatial information. Furthermore, isolating cells from archived samples poses challenges, as artefacts may arise from atypical variations or biases in gene expression data caused by non-biological factors. These limitations can result in misinterpretations of cell-to-cell communication and structural organization, ultimately compromising study accuracy. The lack of spatial information in single-cell sequencing makes it unsuitable for fully understanding the biology of coordinated cellular behaviors within ecological niches comprising several cell types. To address this constraint, recent genomic technologies have introduced various methodologies designed to quantify gene expression within intact tissues, collectively referred to as spatial transcriptomics (ST). ST is a novel technology that enables the direct quantification of RNA expression in tissue slices, providing insights into the spatial distribution of gene expression while maintaining the structural integrity of the tissue. ST technologies are classified into two primary categories: sequencing-based and imaging-based. By elucidating the mechanisms that regulate gene expression alterations among cell types influenced by their spatial proximity, ST offers valuable data on intercellular communication (Piñeiro et al., 2022; Tian et al., 2023). The integration of scRNA-seq and ST technologies enhances the understanding of the onset and progression of skin aging.

#### 4.1.2 Single-cell RNA sequencing and skin aging

Keratinocytes, the primary cell type in the epidermis, play a crucial role in forming a physical barrier for the skin and maintaining homeostasis. However, their gene expression patterns undergo significant changes with age. By employing scRNA-seq technology, researchers have identified these alterations at a single-cell level, revealing transcriptomic heterogeneity among several keratinocyte subpopulations during aging. Research indicates that UVR can induce excessive ROS production in the skin, leading to an overabundance of free radicals that activate the nuclear factor-kappa B (NF-κB) signaling pathway. This activation results in elevated tumor necrosis factor-α (TNF-α) levels and increased expression of matrix metalloproteinases (MMPs), contributing to ECM disintegration and accelerating skin aging. Additionally, NF-κB activation results in its dissociation from the p50/p65/i-κB heterotrimer, allowing it to translocate into the cell nucleus via the nuclear pore complex. Once inside, NF-κB binds to specific DNA sequences, thereby regulating the transcription of target genes and promoting the production and release of inflammatory cytokines such as TNF-α, interleukin (IL)-2, IL-6, and interferon-gamma (INF-γ). These cytokines, in turn, can further reactivate NF-κB, initiating cascade reactions that cause inflammatory tissue damage (Wang Y. et al., 2019; Zou et al., 2021) (Figure 1). Furthermore, Wang et al. constructed a regulatory network using scRNA-seq data from skin samples across different ages, revealing significant transcriptomic alterations in basal cells, spinous cells, mitotic cells, and fibroblasts associated with aging, keratinization, and senescent skin. Their findings indicated a decline in the expression of TFs CCCTC-binding factor (CTCF) and RAD21 cohesin complex component (RAD21) in aging cells. In senescent cells, accumulated DNA damage can activate the tumor protein 53 (p53) signaling pathway, leading to the suppression of CTCF expression. Concurrently, it may also activate the ataxia telangiectasia mutated/ataxia telangiectasia and Rad3-related signaling pathway, inhibiting the activity of cyclin-dependent kinase 1 and cell division cycle 25C and resulting in reduced RAD21 expression, thereby affecting key cellular processes. As the cell cycle progresses, these changes drive cells toward a senescent state. Therefore, the downregulation of the expression of these TFs may replicate the mechanisms underlying cellular senescence (Schepps et al., 2024).

#### 4.1.3 Spatial transcriptomics and skin aging

Similar to scRNA-seq, ST enhances the understanding of cellular and molecular mechanisms associated with human skin aging and regeneration. Both technologies have been employed to investigate epidermal and dermal aging, with a particular focus on alterations in melanocytes and fibroblasts. SenSkin™, a skin-specific cellular senescence gene set, was analyzed and found to be highly expressed in photoaged, naturally aged, and non-replicating CDKN1A+ (p21) cells. This discovery provides a means to identify age-related skin dysfunction by detecting cellular senescence phenotypes while investigating the mechanisms underlying senescent epidermal melanocytes and fibroblasts in the dermal reticular layer during natural skin aging (Ahlers et al., 2022). Consequently, ST is crucial for delineating the processes of human skin aging and uncovering its underlying mechanisms.

### 4.2 Research on genes related to skin aging

Skin aging is categorized into endogenous and exogenous types. Endogenous aging results from intrinsic factors, including time, genetics, immunity, endocrinology, accumulation of senescent cells, and the senescence-associated secretory phenotype (SASP). It is primarily characterized by epidermal thinning, skin dryness, fine lines, diminished elasticity, and pigmentation irregularities (Franco et al., 2022). In contrast, exogenous aging presents with epidermal thickening, deeper wrinkles, skin laxity and roughness, pigmentation alterations, and visible blood vessels beneath the skin surface (Zhang and Duan, 2018). Unlike endogenous aging, exogenous aging is primarily influenced by environmental factors such as UVR, air pollution, tobacco smoke, dietary factors, and cosmetics. Among these, UVR is the predominant cause and is commonly referred to as photoaging. The SASP in photoaging organisms can affect not only the skin but also other tissues and organs, thereby facilitating systemic aging and increasing cancer risk. To investigate the transcriptomic regulatory mechanisms underlying skin photoaging, epidermal and dermal samples were obtained from 16 healthy donors, equally distributed between UV-exposed and unexposed skin areas. RNA-seq analysis revealed significant differences in the expression of 430 miRNAs, 168 lncRNAs, and 136 miRNAs between these skin samples. Additionally, the genes kelch-like family member 41, myosin-binding protein C2, and endoplasmic reticulum aminopeptidase 2 were found to be closely associated with skin photoaging, potentially influencing the process by modulating cell proliferation, intercellular adhesion, and metabolic pathways (Zhao et al., 2023; Yan et al., 2023).

Numerous studies have explored alterations in gene expression associated with skin aging. Glass et al. (2013) analyzed age-related gene expression across diverse human tissues, including the skin, and found that most aging-associated alterations in gene expression profiles were tissue-specific. Furthermore, Yang et al. (2015) examined age-related gene expression modifications across several organs; however, their findings indicated that the skin did not exhibit age-related alterations. Similarly, Kaisers et al. (2017) reported no differential expression of age-related genes in skin tissues. Conversely, Kimball et al. (2018) demonstrated that the downregulation of mitochondrial function might be a hallmark of skin aging. The skin aging process is closely associated with oxidative stress mechanisms, which influence the expression of specific genes. Ultraviolet A (UVA) and hydrogen peroxide (H_2_O_2_) contribute to ROS generation, inducing oxidative stress in skin cells. A study assessed the expression of TFs associated with skin aging and their regulation under oxidative stress, employing complementary DNA microarrays. The basal levels and TF expression in fibroblasts from five young females (under 21 years) and five older females (over 50 years) were analyzed following 24-hour exposure to UVA (7 J/cm^2^) and H_2_O_2_ (20 mM). Findings revealed that 22% of gene expression was elevated in the older cohort at baseline levels. Although both extrinsic independent risk factors can result in similar cell death, H_2_O_2_-induced gene expression was more pronounced than that of UVA, with 19.5% of TFs showing elevated expression in response to H_2_O_2_, compared to only 4% upregulated by UVA (Cho et al., 2018; Hazane-Puch et al., 2010).

### 4.3 Transcriptomics and aging biomarkers

Recent advancements in high-throughput transcriptome sequencing technology have facilitated the identification of biomarkers associated with human skin aging through transcriptomic analyses. circRNAs are crucial regulators of gene expression at both transcriptional and post-transcriptional levels. However, the biological significance of circRNAs in skin aging remain elusive, necessitating further investigation into their mechanism of action. A study analyzing the differential expression of circRNAs in eyelid tissue samples from young and older donors identified several circRNAs linked to skin aging, including hsa-circ_0137613, hsa-circ_0077605, hsa-circ_0003803, hsa-circ_0113488, and hsa-circ_0112861 (Wang et al., 2021). Growing evidence suggests that certain miRNAs play a crucial role in skin aging. For instance, miR-34b-5p expression is elevated in the dermis of aging individuals compared to that of the young group, and its overexpression inhibits collagen and elastin synthesis (Li et al., 2016). Moreover, miR-377 expression is markedly upregulated in passaged senescent human skin fibroblasts (HSFs) and can expedite HSF senescence by targeting DNA methyltransferases. This study utilized miRNA microarrays to examine miRNA expression profiles in aged and youthful skin. A total of 9 dysregulated miRNAs were identified in the aging skin, and the expression levels of miR-302b-3p were assessed. The results revealed that miR-302b-3p activates the c-Jun N-terminal kinase (JNK) signaling pathway by targeting JNK2, thereby amplifying the cellular stress response, which exacerbates damage and aging in skin fibroblasts. Additionally, miR-302b-3p may inhibit gene expression by targeting antioxidant enzyme genes, such as superoxide dismutase 2 and catalase. This inhibition reduces the antioxidant capacity of cells, leading to an accumulation of ROS, causing further damage to cellular components and expediting cellular aging (Tan et al., 2020). EvaRoig-Rosello et al. performed NanoString transcriptome analysis on skin samples across age cohorts, demonstrating that mRNA abundance of the Krüppel-like factor 4 (KLF4) gene progressively increased with aging. KLF4 upregulation inhibits the YAP/TAZ-TEAD pathway, resulting in diminished proliferative capacity of basal progenitor cells. This consequently drives epidermal thinning, barrier dysfunction, and impaired tissue repair (Roig-Rosello et al., 2024).

Skin aging may be associated with specific molecular targets, and transcriptomics, when integrated with other methodologies, provides valuable insights into this process. Studies on extensive bioinformatics analyses and experimental verification have shown that carboxypeptidase E (CPE) is a promising biomarker and potential therapeutic target for skin aging. This study demonstrated that CPE levels are downregulated during replication phases in UVA- and H_2_O_2_-induced dermal fibroblasts. Furthermore, short hairpin RNA-induced CPE knockdown induces aging in HDFs, whereas CPE overexpression delays the aging process. Downregulated CPE levels inhibits collagen synthesis and exacerbates inflammation, highlighting its potential as a therapeutic target for skin aging (Peng et al., 2024). Additionally, the AMP-activated protein kinase (AMPK) signaling pathway offers a theoretical foundation for the development of novel skin anti-aging treatments. Resveratrol, a natural polyphenol, has been shown to promote autophagy, reduce ROS generation, inhibit apoptosis, and prevent cell cycle arrest, thereby mitigating UVA-induced skin photoaging. UVA is the primary cause of skin photoaging, leading to increased skin wrinkles, pigmentation, and laxity. UVA exposure can inhibit autophagy in skin fibroblasts, produce excessive ROS, promote the release of inflammatory mediators and MMPs, and accelerate the breakdown of collagen fibers and the ECM. Autophagy is a cellular self-protective mechanism that involves the breakdown of old or damaged organelles and macromolecular proteins, thereby maintaining cellular homeostasis. This process is essential for delaying the aging process. The AMPK signaling pathway is crucial for regulating autophagy, whereas resveratrol, a natural autophagy regulator, exhibits anti-inflammatory and antioxidant properties. This study provides a theoretical framework for developing new anti-aging treatment strategies and identifies potential therapeutic targets for the future advancement of anti-aging medications (Xia et al., 2024).

### 4.4 Transcriptomics and growth control transcription factors

Transcriptomic analyses are actively exploring the role of growth-regulatory TFs in skin aging. TFs regulate the expression of target genes associated with various cellular and molecular processes, including NF-κB, p53, AP-1, and forkhead box O. One study used dog skin samples, which share structural and functional similarities with human skin, to investigate transcriptional changes associated with aging. Through transcriptome sequencing and bioinformatics analysis, gene expression profiles linked to skin aging were identified, highlighting CTCF and RAD21 as key TFs with downregulated expression in aging dog skin (Wang et al., 2023). Furthermore, another study analyzed eyelid skin samples from healthy female volunteers of various ages using single-cell transcriptome sequencing and bioinformatics analysis. This process resulted in the identification of 11 distinct skin cell types, each with distinct gene expression profiles. Moreover, human epidermal basal cells were categorized into six subpopulations of either quiescent or proliferative cells based on hierarchical transcriptional profiles. Fundamentally, the premature downregulation of the expression of growth control TFs, particularly hairy and enhancer of split-1 (HES1) in fibroblasts and Krüppel-like factor 6 (KLF6) in basal cells, underlies the distinct transcriptional alterations occurring at the cellular level. HES1 and KLF6 inactivation induces cellular senescence. However, the stimulation of HES1 has been demonstrated to inhibit senescence in cutaneous fibroblasts (Chang et al., 2013).

### 4.5 Transcriptomics validates new treatments for skin aging

The primary objective of enhancing skin photoaging is to promote skin health and mitigate both cutaneous and systemic aging. Therefore, exploring novel therapeutics for treating skin photoaging is essential. A study employed 3′ end-sequencing expression quantification (“3-seq”) to analyze gene expression patterns associated with aging, including skin photoaging, as well as to assess the effects of broad-band light (BBL) treatment. The findings indicated that skin aging was associated with notable alterations in the expression levels of 2,265 coding and non-coding RNAs. Among these, 1,293 RNAs exhibited a “rejuvenation” effect following BBL treatment, with their expression levels returning to those characteristic of youthful skin, suggesting a potential role for BBL treatment in skin rejuvenation (Liu et al., 2022). Moreover, bakuchiol, a functional molecule similar to retinol, exhibits considerable antiaging effects. Its gene expression profile closely mirrors that of retinol, particularly in regulating collagen protein synthesis and genes associated with the ECM. Bakuchiol enhances skin aging by activating collagen synthesis pathways in fibroblasts. The precise mechanisms underlying this process are as follows: activation of the transforming growth factor-beta signaling pathway induces the phosphorylation of Smad2/3, subsequently enhancing the production of type I collagen. Given that type I collagen is the primary structural protein in the skin, its augmented synthesis contributes to improved suppleness and firmness. Simultaneously, inhibiting the NF-κB signaling pathway reduces the production of MMP1 and MMP3, thereby limiting collagen breakdown. This dual mode of action helps maintain collagen equilibrium in the skin and delays the aging process. Clinical research demonstrated that after 12 weeks of using skincare products containing 0.5% bakuchiol, participants exhibited significant improvements in fine lines, wrinkles, pigmentation, and elasticity. Notably, these benefits were achieved without the common side effects associated with conventional retinol therapy. This study establishes a scientific foundation for the potential use of bakuchiol as a novel anti-aging agent (Chaudhuri and Bojanowski, 2014).

## 5 Proteomics

Proteomics is the study of the expression, structure, function, and post-translational changes of all proteins produced by living cells. This field encompasses protein identification, post-translational modification identification, protein complex characterization, comparative proteomics, modification genomics, glycomics, and targeted proteomics (Al-Amrani et al., 2021). Currently, proteomics is extensively applied to investigate the molecular basis of diseases and identify potential biomarkers and therapeutic targets. These studies primarily utilize mass spectrometry (MS), two-dimensional electrophoresis, and bioinformatics (Zhang et al., 2014).

### 5.1 High-throughput proteomics

MS-based high-throughput proteomics is an essential tool for comprehensive protein characterization. These approaches aim to reduce sample analysis time while enhancing proteome coverage depth, addressing the vast complexity of the proteome (Wang PW. et al., 2019).

To investigate skin photoaging and the mechanisms activated by UVR at varying wavelengths, Wang et al. conducted a proteomic study on mouse skin samples exposed to UVA (320–400 nm) and UVB (290–320 nm). The target proteins were subsequently validated using immunohistochemistry. The study revealed a signification upregulation of MMP expression in UVA-irradiated skin; however, UVB-irradiated skin exhibited only a marginal increase in MMP levels compared to control samples. A total of 17 key proteins were identified, which are involved in cell growth control, apoptosis, inflammation, protein folding, and endoplasmic reticulum stress—all of which may contribute to photoaging in UVA- and UVB-exposed skin (Wang PW. et al., 2019). Additionally, research suggests that heat shock protein 27 (HSP27) is significantly associated with skin cell differentiation and proliferation. In this study, HSP27 expression increased by 4.52-fold in the UVA-exposed group and 1.86-fold in the UVB-exposed group, suggesting a stronger correlation with cell differentiation in the UVA group than in the UVB group. Furthermore, UVB-exposed skin exhibited a notable increase in the expression of proliferating cell nuclear antigens, indicating that UVB radiation primarily stimulates keratinocyte proliferation rather than differentiation (Laimer et al., 2010).

A high-throughput proteomic analysis conducted by Aimer et al. on foreskin samples from 10 young and 10 older males identified 30 proteins exhibiting differential expression exceeding 1.3-fold in relation to endogenous skin aging. Among these, 12 proteins exhibited downregulated expression levels, whereas 18 demonstrated upregulated expression, with these expressions linked to the onset of aging. The study highlighted that the decrease in the levels of collagen types I and III is a hallmark of aging skin. Specifically, the observed downregulation of collagen type I (COL1A1) expression at both genomic and proteomic levels may be attributed to oxidative stress and mitochondrial mutations resulting from aerobic cellular metabolism (Gromov et al., 2003). Additionally, novel proteins associated with skin aging were identified, including phosphatidylethanolamine-binding protein (PEBP) and carbonic anhydrase. Moreover, keratin 5 (KRT5) was recognized as a new housekeeping protein in the aging process, suggesting its potential use as a normalization criterion for expression levels in future skin aging studies. The analysis also revealed a collection of proteins—KRT9, KRT4, KRT2, osteoglycin, tropomyosin 2, actin alpha cardiac muscle 1, and COL1A1—which, despite their diverse molecular structures, share a common biological function. These proteins may serve as potential indicators or controls in future studies on skin aging (Gromov et al., 2003).

Gromov et al. conducted a high-throughput proteomic analysis of epidermal samples from 20 pairs of young (21–30 years) and older (75–92 years) individuals, identifying 22 proteins with differential expression exceeding 1.5-fold during the skin aging process. Among these, the expression of 16 was upregulated, whereas that of 6 was downregulated, including manganese superoxide dismutase (Mn-SOD), the p85 subunit of PI3K, and proteasome activator complex subunit 3 (PA28-α). Additionally, the study identified a cohort of six peptides (Mn-SOD, p85 subunit of PI3K, PA28-α, SSP 0107, myxovirus resistance protein A, tryptophan-tRNA synthetase), which serve as markers of proteins synthesized in response to INF-γ in primary human keratinocytes, predominantly located in aged epidermis. The levels of these peptides were upregulated in the epidermis, offering novel insights into the immunoregulatory mechanisms associated with skin aging (Cheung and Juan, 2017). Consequently, high-throughput proteomics proves valuable in elucidating protein interactions and identifying critical targets and pathways.

### 5.2 Quantitative proteomics

Quantitative proteomics specifically identifies protein species and quantities while quantifying differences between healthy and diseased groups, thus generating taxonomic models that yield critical insights into molecular interactions, key signaling pathways, and biomarker screening (Zhang et al., 2023).

Gromov employed data-independent acquisition (DIA) proteomics technology for quantitative proteomic analysis to non-invasively obtain epidermal proteins from healthy Chinese individuals across various ages and sexes. A total of 1,270 unique proteins were identified, with 95 exhibiting differential expression among age groups; the expression of 38 was downregulated, whereas that of 57 were upregulated. Among the identified proteins, ABRAXAS sister of BRCA1 A complex subunit 2, vesicle amine transport 1, and galectin-3 binding protein emerged as potential aging biomarkers, exhibiting differential expression in the young cohort of varying sexes, but not in the aging cohort. These results suggest their potential as distinguishing biomarkers for the aging process in males and females. The differentially expressed proteins were associated with various protein-protein interaction (PPI) networks linked to the Kyoto Encyclopedia of Genes and Genomes (KEGG) pathway. These proteins were notably enriched in the platelet activation, complement, coagulation cascade, and vascular endothelial growth factor pathways. Copper-cyanoplasmin is particularly noteworthy because of its association with aging. It has 16 adjacent proteins in the PPI network, participates in the iron death metabolic pathway, and increases in expression with age, correlating with sex differences (Xu et al., 2024). DIA technology has demonstrated strong capabilities in quantitative proteomics research, elucidating significant age- and sex-related aging biomarkers while also offering insights into protein interactions within intricate biological pathways, thereby establishing a crucial foundation for a deeper understanding of the mechanisms underlying skin aging. Xu et al. utilized Isobaric Tag for Relative and Absolute Quantitation technology to analyze skin samples from D-galactose-induced senescent animals treated with NTP (silver fungus-derived polysaccharide) using DIA technology. The analysis identified 35,093 peptides and 4,646 proteins. Among these, 43 proteins exhibited differential expression exceeding 1.3-fold following NTP treatment, with 23 upregulated and 20 downregulated. The differentially expressed proteins were found to participate in several biological functions. Gene Ontology (GO) analysis linked them primarily to cellular and metabolic regulation, immune system responses, and structural components. Additionally, KEGG pathway enrichment analysis indicated their involvement with glycolysis, gluconeogenesis, nucleotide metabolism, ECM-receptor interactions, and other related processes (An et al., 2020).

### 5.3 Bioinformatics analysis

To comprehensively understand the differential expression of cellular proteins and their biological relevance under both normal and pathological conditions, bioinformatics analysis serves as a powerful data-mining instrument. It facilitates protein functional annotation, elucidation of novel molecular interactions, and investigation of protein-module and complex interactions. Additionally, it aids in the investigation of disease pathways by analyzing large-scale proteomics datasets (Zhang et al., 2023).

An et al. employed phosphorylated protein array experiments to examine normal HDFs induced into quiescent, senescent, and proliferative states. They collected data on intracellular phosphorylated protein species and expression levels, constructed a Boolean network model, and conducted simulations to analyze cellular senescence signal transduction. By simulating the network’s dynamic evolution and optimizing the model’s logic rules using an evolutionary method, the study identified 3-phosphoinositide-dependent protein kinase 1 as a pivotal target for cellular senescence, capable of converting cells from a senescent state to a quiescent state (Zhang et al., 2024).

Zhang et al. utilized 4D-lable free proteomics to conduct a quantitative analysis of mouse skin samples, comparing an experimental group treated with CASIN—a Cdc42-specific inhibitor—to an untreated control group across different age cohorts. A total of 6,672 proteins were identified, of which 1,015 were categorized into 10 clusters. The Mfuzz clustering approach was applied to analyze GO functions, KEGG pathways, and associated protein structural domains. The findings indicated that CASIN demonstrated considerable anti-aging effects on proteins associated with Cluster 9, which are primarily linked to ribosomal function. PPI network analysis of 11 ribosome-associated proteins between the WT9-N and WT15-N groups of Cluster 9 was conducted using the STRING database. The findings suggested that the anti-aging effects of CASIN were facilitated by 60S ribosomal protein L4 (RPL4), which played a role in collagen synthesis and influenced fibroblast cytoskeletal morphology (Haydont et al., 2019).

## 6 Metabolomics

Metabolomics utilizes nuclear magnetic resonance, MS, and other technologies to elucidate metabolic network dynamics within the body. By analyzing alterations in various humoral metabolites secreted in response to external interventions, metabolomics provides a holistic, dynamic, integrated, and analytical perspective. This approach presents a novel strategy for studying skin aging (Mishur and Rea, 2012).

### 6.1 Qualitative and quantitative metabolite analyses

Metabolomics enables both qualitative and quantitative assessments of metabolites in aged skin, offering insights into cellular functions and the effect of internal and external stimuli. Numerous studies have examined skin samples using gas chromatography (GC)-MS and liquid chromatography-MS. Findings indicate that ceramide levels may decline in lipid metabolism, whereas advanced glycosylation end products tend to increase in glucose metabolism with skin aging, indicating a pattern of metabolic alterations associated with skin aging (Azma et al., 2019). The gas chromatography time-of-flight mass spectrometry (GC-TOF/MS) technique was used to investigate the effects of camellia seed crude oil and *Camellia sinensis* emollient oil on the skin of D-galactose-induced aging mice (Li et al., 2023). The study identified nine distinct metabolites between the normal and model groups, one metabolite between the model and camellia seed crude oil groups, and eight metabolites in the model group, shared by both the *C. sinensis* emollient oil and vitamin E groups. The analysis of metabolic pathways highlighted three key pathways—alanine, aspartate, and glutamate metabolism—which are linked to oxidative stress and have significant biological effects (Fredman et al., 2022). Based on these findings, camellia oil is hypothesized to mitigate skin photoaging by regulating oxidative stress-related metabolic pathways. Furthermore, research has identified differential metabolites in patients with neurosyphilis, atopic dermatitis, and DOCK8 deficiency, offering new insights for disease diagnosis and the discovery of new therapeutic targets (Tabula Muris Consortium, 2020). Alterations in gene expression may significantly influence skin aging by affecting metabolic pathways within skin cells (Schaum et al., 2020; Farage et al., 2008). Overall, the integration of macro-genomics, transcriptomics, and metabolomics holds promise for advancing the understanding of aging mechanisms.

### 6.2 Pathology of skin aging and aging

The rate of intrinsic skin aging varies among individuals and different body regions, influenced by factors such as ethnicity and hormone fluctuations (Ndiaye et al., 2014). Conversely, extrinsic skin aging is primarily driven by environmental factors, with UVR resulting in elevated amounts of ROS (Gu et al., 2020) in the skin. This excessive ROS accumulation leads to oxidative stress, which disrupts metabolic pathways, ultimately contributing to wrinkles and other symptoms of aging (Mai et al., 2019). At the microscopic level, skin aging is characterized by structural and functional alterations in the dermis, a reduction in skin thickness, modifications in ECM composition, and impaired fibroblast function. These pathogenic characteristics are intricately associated with metabolomic alterations, and metabolomic investigations are expected to uncover aging-related biomarkers (Mishur and Rea, 2012).

### 6.3 Study of key metabolic pathways and metabolic networks in skin aging

Metabolomics enables the qualitative and quantitative assessments of small-molecule metabolites in dermatological disorders while also facilitating the construction of metabolic networks. A study examining the effects of Dendrobium bulb on the skin of D-galactose-induced aging mice using GC-TOF/MS identified nine distinct metabolites that differentiated the normal group from the model group (Fu et al., 2022). Additionally, unique metabolites were identified between the model and bulb groups, implicating three primary metabolic pathways: amino acid metabolism, glucose metabolism, and the flavonoid pathway. It was hypothesized that the Dendrobium bulb may mitigate skin photodamage by modulating oxidative stress-related metabolic pathways. Similarly, procambium has been suggested to mitigate skin photoaging by modulating oxidative stress-related metabolic pathways. A study investigating the intervention mechanism of Xijinwan in skin photoaging in rats using GC-MS metabolomics technology (Chong et al., 2021) identified six key metabolites significantly associated with photoaging. These metabolites were linked to glutathione metabolism, arginine and proline metabolism, and arachidonic acid metabolism, offering insights into the pathogenesis of skin photoaging and the mechanism of action of Xijinwan. A GC-MS-based metabolomics analysis further identified 716 metabolites in the human body, with certain metabolites exhibiting age-related upregulation or downregulation, significantly affecting human physiology. Notably, N-acetylglutamate in metabolites may serve as an indicator of skin cell energy metabolism and subsequent adaptive physiological changes. Additionally, the porphyrin production pathway significantly influences skin aging by modulating the oxidative-reductive system, thereby influencing dermal senescence (Katsuumi et al., 2024).

### 6.4 Use of metabolomics to delay skin aging

Metabolomics research has been extensively employed to develop anti-aging medicines. Pharmacologically, sodium-glucose cotransporter 2 inhibitors, such as cagliflozin, have been shown to alleviate adipose tissue dysfunction and extend longevity in prematurely aged mice (Coletti et al., 2022). In terms of lifestyle interventions, high-intensity interval exercise has been found to enhance mitochondrial activity, whereas calorie-restricted diets have been demonstrated to contribute to slowing the aging process (Chong et al., 2021). Additionally, Traditional Chinese medicine therapies, including single-herb treatments, such as *Scutellaria baicalensis*, and compound herbal formulations, including Er Zhi Wan, have been demonstrated to improve metabolic disorders by regulating metabolite levels and exerting multi-target effects on the metabolic network, ultimately contributing to the slowing of skin aging (Yun et al., 2022).

## 7 Summary and outlook

Current skin aging research encompasses various topics, including skin microbiota, the skin-gut axis, DNA methylation, miRNA, RNA sequencing, quantitative proteomics, and quantitative metabolite assays. The incorporation of multi-omics analysis strategies enables a more comprehensive understanding of the mechanisms underlying gene regulation, epigenetics, molecular-cellular pathways, aging markers, protein function, metabolites, and therapeutic targets associated with skin aging. This holistic approach enhances clarity regarding the etiology and pathogenesis of skin aging. The implementation of this comprehensive research framework can aid in the identification of biomarkers and therapeutic targets, while also establishing a solid scientific foundation for developing anti-aging medications. Each analytical method has unique advantages and disadvantages; consequently, the combined use of multi-omics technologies allows for mutual validation and refinement, representing the future trajectory of methodological advancements in aging research.

It should be noted that, due to space limitations and the focused scope of this review, the multi-omics studies presented herein aim to exemplify methodological applications and findings, rather than providing an exhaustive compilation of all relevant literature. Representative or landmark studies were selected for discussion.

## Glossary

AGEs: advanced glycosylation end products
AKT: protein kinase B
BBL: broadband light
BCL2: B-cell lymphoma 2
BAX: BCL2-associated X protein
CCL2: C-C motif chemokine ligand 2
CDKN2A: cyclin-dependent kinase inhibitor 2A
COL1A1: collagen type I alpha 1 chain
COL3A1: collagen type III alpha 1 chain
CTCF: CCCTC binding factor
CXCL8: C-X-C motif chemokine ligand 8
DHM: dihydromyricetin
DHMT1: DNA methyltransferase 1
FOXO3: forkhead box O3
GC-MS: gas chromatography-mass spectrometry
GC-TOF/MS: gas chromatography time-of-flight mass spectrometry
IL-1β: interleukin 1 beta
IL-6: interleukin 6
LC-MS: liquid chromatography-mass spectrometry
MMP1: matrix metalloproteinase 1
MMP3: matrix metalloproteinase 3
MMP9: matrix metalloproteinase 9
miRNA: microRNA
NMR: nuclear magnetic resonance
Nrf2: nuclear factor erythroid 2-related factor 2
RNA-seq: RNA sequencing
ROS: reactive oxygen species
SIRT1: sirtuin 1
SGLT2: sodium-glucose cotransporter 2
SOD2: superoxide dismutase 2
TFs: transcription factors
TNF-α: tumor necrosis factor alpha
UVA: ultraviolet A radiation
